# Injury as a stressor in 67 first year contemporary dance students: a longitudinal cohort study

**DOI:** 10.3389/fpsyg.2025.1504292

**Published:** 2025-12-10

**Authors:** Judith-Elisa Kaufmann, Janine H. Stubbe, Rogier M. van Rijn, Rob G. H. H. Nelissen, Maaike G. J. Gademan

**Affiliations:** 1 Department of Orthopaedics, Leiden University Medical Centre, Leiden, Netherlands; 2 Academy for Dance Pedagogy and Dance Medicine, Vienna, Austria; 3 Codarts, Rotterdam, University of the Arts, Rotterdam, Netherlands; 4 PErforming Artist and Athlete Research Lab (PEARL), Rotterdam, Netherlands; 5 Department of Clinical Epidemiology, Leiden University Medical Centre, Leiden, Netherlands

**Keywords:** dancer, contemporary dance, pre-professional, injury, psychological, stress, first injury, subsequent injury

## Abstract

**Introduction:**

In athletes, injuries lead to increased stress but athletes cope better with subsequent than with first-time-injury. In dancers such information is lacking. Thus, we investigated whether injuries are associated with increased stress-levels, and if subsequent injuries are associated with higher perceived stress levels than first injury.

**Methods:**

Bachelor dance students were prospectively followed during five academic years (2016-2022). During the first month of the year, respectively, baseline characteristics were recorded using the “Performing artist and Athlete Health Monitor” (PAHM) including the VAS pain scale, the VAS stress scale, and the “Oslo Sport Trauma Research Centre Questionnaire on Health Problems” (OSTRC). Throughout the year, stress, injuries and injury severity were reported monthly. The first-month assessment served as baseline in which dancers had to be injury free to be included in this analysis. Paired t-tests were conducted.

**Results:**

67 dancers (age 18.8 ±.8 years, 58.2% female) sustained at least one injury, of those 33 at least one subsequent injury. OSTRC severity scores ranged from a mean of 28.7 ±17.5 for first injuries to 31.4 ±17.1 for subsequent injuries. The mean baseline stress level for injuries was 24.3 ±17.2. Stress levels during first and subsequent injuries were elevated compared to the baseline stress levels, 34.8 ± 18.7 and 35.1 ±23.0, respectively. There were no differences between stress levels during first and subsequent injuries.

**Discussion:**

Injuries are associated with increased stress levels in dancers, but stress levels were equally high after first and subsequent injury. We emphasize the importance of a holistic approach to injury rehabilitation, thus not only focusing on the physiological injury as such, but also include the dancers’ psychological recovery and a focus on stress management for both enhanced recovery as well as prevention of future injuries.

## 1 Introduction

It is well known that mental stress has negative effects on health. For instance, chronic mental stress has been linked to several diseases, including mental disorders (Calcia et al., 2016; Steptoe, 1991; Dorian and Garfinkel, 1987; Salleh, 2008; Gerber et al., 2018). According to Andersen and Williams’ stress-injury model, mental stress increases the risk of injury by inducing neurophysiological changes which can lead to a loss of neuromuscular control and flexibility, increased muscular tension, restriction of peripheral vision, or attentional deficits (Andersen and Williams, 1988).

In studies on ballet dancers the association between general as well as dance-related stress and injuries was confirmed (Mainwaring and Krasnow, 1999; Noh et al., 2005; Adam et al., 2004). Recently, research has demonstrated that this association could also be found in contemporary dancers (van Winden et al., 2021). Moreover, Noh and colleagues showed that being free from worry and being self-confident reduced the number of injuries, whilst dancers who had negative dance stress (i.e., presence of worries) had to face longer duration of injuries (Noh et al., 2005).

Prior research suggests that stress increases the risk of injury (Noh et al., 2005; Adam et al., 2004; van Winden et al., 2021; Mainwaring, 1999). However, the injury as such might be regarded as a stressor as well. Several models attempted to describe stress responses to injury. Brewer’s response model suggests that injury is first appraised cognitively, influenced by personal and situational factors, followed by an emotional and behavioral stress response (Brewer, 1994). Mainwaring’s model highlights a more complex, multidimensional, person-to-situation interaction between factors related to the affected individual and their environment rather than a linear process of events (Mainwaring, 1999). Therein, the individual’s psychological, physical, behavioral and social responses act as moderators, affecting and being affected by intrinsic determinants (e.g., demographics, traits, coping behavior, identity, nutritional aspects, knowledge, other stressors), as well as extrinsic factors (e.g., dance environment, financial situation, social support, dance culture and identity, treatment) (Mainwaring et al., 2001). As such, Mainwaring’s model stresses the importance of a more holistic approach to the individual who sustained an injury. The latter is important since it will affect not only the physical rehabilitation of the injured but also address the individual athlete’s psychological recovery which requires strong social support (Mainwaring, 1999; Lu and Hsu, 2013).

In sports science it has been described that athletes cope better with subsequent injuries than with a first-time-injury, having shown to be less anxious and more accepting with subsequent injury (Johnson, 1996). In dancers, however, such information is lacking. A very common approach in traditional dance settings is to perform and train despite the injury and in presence of pain (Jenkins et al., 2013; Wainwright et al., 2005; Harrison and Ruddock-Hudson, 2017). The latter has also been described in other athletes (Weinberg et al., 2013). Such an approach after a sustained injury may intensify dancers’ fear of more severe and/or subsequent injuries (Wainwright et al., 2005; Tajetfoxell and Rose, 1995), which might increase stress levels after subsequent injuries. Therefore, we hypothesize that dancers’ stress levels upon second- or further injuries are increased instead of decreased.

For that matter, the purposes of this study were to investigate whether (1) injuries are associated with increased stress levels, and (2) subsequent injuries are associated with higher perceived stress levels than first injury in first-year contemporary dance students.

## 2 Methods

### 2.1 Participants

From the study year 2016–2017 until 2021–2022, five cohorts of first-year contemporary dance students (*N* = 145) of Codarts University of the Arts, the Netherlands, were prospectively followed during one academic year (September to June). Results from the study year 2019–2020 were excluded from the analysis due to COVID-19 restrictions which prevented students from following regular classes.

The participants were enrolled in a four-year Bachelor of Dance program, which focuses on acquiring a wide range of modern dance techniques and competencies in various modern and contemporary styles as well as (modern) jazz, ballet, and ‘floorwork’. Furthermore, the first-year of the Bachelor of Dance’s curriculum focuses on performance creative skills (i.e., improvisation, composition, and drama) as well as on basic knowledge on health (i.e., nutrition, anatomy, and sport psychology). First year dance students were included in this study if they were injury free in the first month of the academic year (to obtain a baseline stress level) and had sustained at least one injury in the rest of the academic year. An intake assessment as well as a baseline stress measurement had to be available. Injury-free dancers were included in the analysis to investigate seasonal effects.

### 2.2 Procedures

Data were collected at regular intervals for management and educational purposes and data collection was additionally embedded in the curriculum. In accordance with the Declaration of Helsinki, all students were informed prior to the procedure and provided written consent. The Medical Ethics Committee Erasmus MC of Rotterdam, the Netherlands, granted ethical approval for the study (MEC-2019-0163).

During the first month of each academic year, baseline characteristics were recorded, e.g., age (years), sex (male/female) and one-year history of injury (yes/no). Injuries in the past year before students entered the educational program at Codarts Rotterdam were registered. This one-year injury history was defined as “any physical complaint resulting in a fulltime loss of dance activities (e.g., participation in class, rehearsal, performance) for at least 1 week beyond the day of onset in the past year” (van Seters et al., 2020). Throughout the academic year, the students were asked to complete monthly questionnaires on their physical and mental health through the “Performing artist and Athlete Health Monitor” (PAHM). PAHM was developed by Codarts Rotterdam and is used to monitor physical and mental health in pre-professional and professional performing artists and athletes (Stubbe et al., 2018; Karreman et al., 2019; van Winden et al., 2019). This online monitoring tool consists of several questionnaires and items, e.g., the visual analogue scale (VAS) pain scale, the VAS stress scale, the “Oslo Sport Trauma Research Center Questionnaire on Health Problems (OSTRC),” and items on injury characteristics, sleep quality, feelings, emotions, satisfaction with rehearsals and satisfaction with performances.

### 2.3 Assessment of injury

The “Oslo Sport Trauma Research Center (OSTRC) Questionnaire on Health Problems” was used during the monthly questionnaire. It consists of four key questions on the consequences of health problems on dance participation, training volume, performance and the degree to which students perceive any symptoms (Clarsen et al., 2014). Possible answers ranged from 0 (no problem, no reduction, no effect, or no symptoms) to 25 (cannot participate at all or severe symptoms; Clarsen et al., 2013). Questions 1 and 4 were scored on a four-point scale (0, 8, 17, and 25), while questions 2 and 3 were scored on a five-point scale (0, 6, 13, 19, and 25). The OSTRC Questionnaire has a high internal consistency, with a Cronbach’s alpha of 0.96, good face validity (Clarsen et al., 2014; Clarsen et al., 2013), and has previously been used within the performing arts (van Winden et al., 2021; van Seters et al., 2020; Stubbe et al., 2018; van Winden et al., 2019; Kenny et al., 2018; Stubbe et al., 2021; Kaufmann et al., 2021; van Winden et al., 2020a; van Winden et al., 2020b).

The severity of a health problem was calculated using the sum score of the four questions (scale 0–100) according to the method proposed by Clarsen et al. (2013). If the severity score was higher than zero, a health problem was registered and the student was asked whether the health problem was an injury, mental complaint, or other problem. An all-complaint injury (i.e., substantial or non-substantial injury) was defined as “any physical complaint sustained by a dancer resulting in a severity score higher than zero (i.e., leading to consequences on participation, training volume, and/or performance), irrespective of the need for medical attention or time-loss from dance activities.” (van Winden et al., 2019) Substantial injuries were defined as problems leading to moderate or severe reductions in training volume (value ≥13 on question 2 of the OSTRC Questionnaire) or moderate, severe, or complete reductions in performance (value ≥13 on question 3 of the OSTRC Questionnaire; Clarsen et al., 2014). Non-substantial injuries were defined as problems requiring no or little reduction in training volume (value <13 on question 2 of the OSTRC Questionnaire) or no or little reduction in performance (value <13 on question 3 of the OSTRC Questionnaire; Clarsen et al., 2014). For the current study we selected the first and subsequent injury irrespective of their severity.

We categorized dancers as “dancers with substantial injuries” if they were injury-free in the month of baseline assessment (September) and sustained a substantial injury in the period between October and June. Dancers were categorized as “dancers with all-complaint injuries” if they were injury-free in the month of baseline assessment (September) and sustained a non-substantial or substantial injury in the period between October and June.

Subsequent injuries were considered subsequent if they were new injuries sustained after the first injury, or if they were sustained months apart from the first injury. For the latter, injury data had to be available prior to the injury and the dancers had to be injury-free before the month of the subsequent injury.

### 2.4 Assessment of stress

On a monthly basis, students were asked to indicate their stress levels. Perceived general stress scores were measured using a visual analogue scale (VAS) ranging from 0 (no stress) to 100 (extreme amount of stress). The VAS is frequently used in stress assessment; it also has been used to measure stress in dancers (van Winden et al., 2021), and several validity studies have highlighted its psychometric properties. The VAS is at least as sensitive as other stress scales (i.e., 14-items Perceived Stress Scale; Lesage et al., 2012), is significantly correlated with objective stress measurements such as cardiovascular parameters (e.g., heart rate, blood pressure; Hulsman et al., 2010), and shows satisfactory reliability (Lesage et al., 2009) and inter-judge reliability (Lesage et al., 2011). No minimal clinically important difference has been determined (Rotter et al., 2020).

The stress level assessed in the first month of the academic year (September) was used as the baseline stress level. Stress levels measured within the month in which a first or subsequent injury occurred were used as accompanying injury stress levels. In addition, we subtracted the baseline stress levels from the stress levels that were reported during the month in which the first injury occurred as well as from the stress levels accompanying the subsequent injury to calculate changes in stress levels.

### 2.5 Statistical analysis

SPSS Version 25 (IBM Corp., Armonk, USA) was used for all statistical analyses. Descriptive statistics were applied to describe baseline characteristics and stress scores using medians, interquartile ranges, frequencies and proportions (%). Normality was tested for parametric procedures using the Shapiro–Wilk test as well as explorative descriptive statistics, histograms, and Q-Q-plots. The analyses were conducted for the whole group, i.e., all-complaint injuries, as well as stratified for dancers with substantial injuries and dancers who sustained non-substantial injuries, respectively.

The differences between baseline stress levels and stress levels during injury were calculated with a paired students t-test. Additionally, we compared the changes in stress levels during the first and subsequent injury with a paired students t-test. For the t-tests we performed a complete case analysis (i.e., including those participants for which we have no missing data regarding stress scores).

To explore potential seasonal effects on stress levels, we calculated average monthly stress scores among dancers who remained injury-free throughout the entire year. To enable comparison with injured dancers, we then compared each non-injured dancer’s baseline stress level with their stress level from a randomly selected month. This random selection was designed so that the distribution of selected months matched the monthly distribution of first all-complaint injuries. Each injury-free dancer was therefore assigned 1 month and its corresponding stress score; for instance, dancer 1 was assigned October, and dancer 2 December. This approach ensured that every dancer contributed both a baseline value and a stress score from a single, randomly assigned month, while preserving the temporal pattern of injuries in the sample. We then used paired t-tests to compare baseline stress levels with those from the assigned months.

## 3 Results

### 3.1 Participants

**Figure 1:**
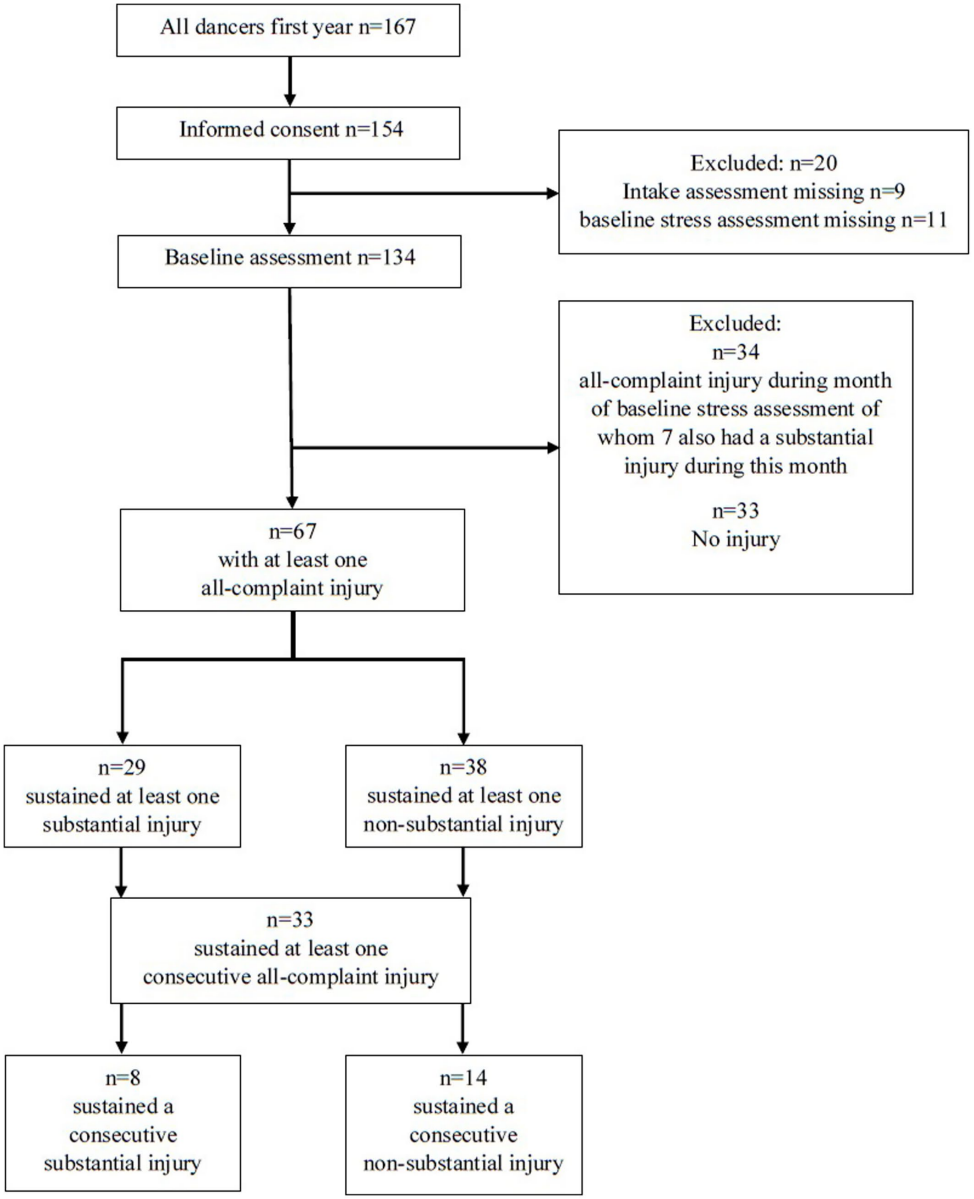
Flowchart documentaion of the inclusion criteria for analysis.

Our study included 67 dancers from 5 annual cohorts of first-year bachelor of dance students. Who were injury free at the baseline assessment and sustained at least one injury during the academic year. Their data was used to answer our primary research question (Figure 1). The 67 included dancers had a mean age of 18.8 ± 0.8 years, and 39 (58%) of the 67 dancers were female. The number of participants in each of the five cohorts we studied in the 5 years were approximately the same size, ranging from 12 to 14 dancers. Demographics can be found in Table 1.

**Table 1:** Demographics: dancers with at least one substantial or all-complaint injury.

Characteristics	*N* = 67
Age at baseline (years)	18.8 ± 0.80
Female	39 (58.2)
Cohort (year of study)	12 (17.9)
2016–2017	14 (20.9)
2017–2018	13 (19.4)
2018–2019	14 (20.9)
2020–2021	14 (20.9)
2021–2022	

Number of substantial injuries per dancer	*N* = 29
1 substantial injury	18 (62.1)
2 substantial injuries	8 (27.6)
3 substantial injuries	1 (3.5)
4 substantial injuries	2 (6.9)

Number of all-complaint injuries	*N* = 67
1 all-complaint injuries	21 (31.3)
2 all-complaint injuries	20 (29.9)
3 all-complaint injuries	13 (19.4)
4 all-complaint injuries	5 (7.5)
5 all-complaint injuries	6 (9.0)
6 all-complaint injuries	1 (1.5)
7 all-complaint injuries	1 (1.5)

The values are given as the mean ±standard deviation, *n* (%) of the population.

### 3.2 Injuries

Throughout all cohorts, 67 dancers reported at least one all-complaint injury (substantial or non-substantial). Of these 67 dancers, 33 had at least one subsequent injury. OSTRC severity scores for first all-complaint injuries showed a mean of 28.7 ± 17.5 (minimum 6 to maximum 74). For subsequent all-complaint injuries OSTRC scores were 29.8 ± 16.3, with a minimum of 6 and a maximum of 67. The number of all-complaint injuries per dancer ranged from 1 to 7. In our population, 29 dancers (43%) sustained at least one substantial injury, of whom 8 also had a subsequent substantial injury. The number of substantial injuries per dancer ranged from 1 to 4 (Table 1). 38 dancers reported at least one non-substantial injury, of whom 14 had a subsequent non-substantial injury (Figure 1).

### 3.3 Stress levels

Mean baseline stress levels of the different groups are depicted in Tables 2, 3. The mean stress level for all included dancers was 26.9 ± 19.8. In the whole group as well in the stratified analysis for substantial injuries and non-substantial injuries, we found that the stress levels during both the first injury and the subsequent injuries were significantly elevated compared to the baseline stress levels (Table 2). The differences ranged from an 8-point increase in the non-substantial injury group during first injury to 27 points in the substantial injury group during both their first and subsequent injury. No differences were found between stress levels during first injuries and subsequent injuries. No statistically significant difference for a seasonal effect was found in the injury free dancers (*n* = 33; mean difference −6.0 ± 23.6; CI95% −14.6 to 2.3).

**Table 2:** Comparison stress levels at baseline to stress levels in the month of an injury occurrence (paired t-tests).

Injuries	Baseline Stress levels	First injury Stress levels	Mean difference between baseline & first injury	Subsequent Injury Stress levels	Mean difference between baseline & subsequent injury	Mean change difference between first & subsequent injury
	Mean (SD)	Mean (SD)	Mean (SD) (95%CI)	Mean (SD)	Mean (SD) (95%CI)	Mean (SD) (95%CI)
All-complaint Injuries *N* = 67	26.9 ± 19.8	35.5 ± 19.7	−8.6 ± 23.4 (−14.3 to −2.9)	NA	NA	NA
All-complaint Injuries *N* = 33	24.3 ± 17.2	34.8 ± 18.7	NA	35.1 ± 23.0	−10.8 ± 26.4 (−20.1 to −1.4)	−3.3 ± 18.3 (−9.8 to 3.2)
Substantial injuries *N* = 29	28.7 ± 21.4	39.4 ± 22.1	−10.7 ± 23.9 (−19.8 to −1.6)	NA	NA	NA
Substantial Injuries *N* = 8	28.0 ± 16.5	49.8 ± 13.1	NA	54.8 ± 25.1	−26.8 ± 24.5 (−47.2 to −6.3)	−5.0 ± 26.0 (−26.8 to 16.8)
Non-substantial Injuries *N* = 38	25.5 ± 18.7	33.7 ± 21.0	−8.2 ± 23.6 (−15.9 to −0.4)	NA	NA	NA
Non-substantial Injuries *N* = 14	22.1 ± 13.7	28.1 ± 18.5	NA	29.8 ± 17.2	−7.7 ± 22.1 (−20.5 to 5.1)	−1.61 ± 19.9 (−13.1 to 9.9)

The values are given as the mean ±standard deviation (SD), *n* (%) of the population; CI: Confidence interval, 95%.

**Table 3:** Stress levels of injury-free and injured dancers, including injury frequencies per month.

Stress levels	Sept	Oct	Nov	Dec	Jan	Feb	Mar	Apr	May
N injury-free	33	31	29	28	23	24	23	22	24
Stress level Mean ±SD	26.9 ± 20.7	34.7 ± 24.3	33.1 ± 20.3	27.8 ± 20.5	30.7 ± 24.0	23.1 ± 22.8	30.0 ± 21.1	39.8 ± 26.4	42.5 ± 26.4
N injured	67	63	61	53	57	56	56	53	50
Stress level Mean ±SD	26.9 ± 19.8	28.8 ± 18.2	32.7 ± 18.5	30.3 ± 20.6	31.4 ± 19.2	33.7 ± 21.5	38.6 ± 22.5	41.8 ± 24.6	47.3 ± 23.8
Injuries *n* (%)									
First all-complaint injury	NA	16 (23.9)	21 (31.3)	4 (6.0)	8 (11.9)	7 (10.4)	7 (10.4)	4 (6.0)	-
subsequent all-complaint injury	NA	-	7 (10.4)	11 (16.4)	8 (11.9)	6 (9.0)	7 (10.4)	4 (6.0)	3 (4.5)

Values are given as *N* of the injury-free and all-injury population, mean ±standard deviation (SD).

## 4 Discussion

In first year contemporary dance students, we prospectively investigated whether injuries are associated with increased stress levels, and if subsequent injuries are associated to higher perceived stress levels than first injuries. Our results showed that stress levels increased during a period when an injury was sustained. However, the stress levels during subsequent injury and the stress levels perceived during the period of the first injury seemed comparable.

Research has shown that the perception of stress is subjective, depending on factors such as the personality and coping behavior of the injured person, the motivational climate or perceived social support (Adam et al., 2004; Mainwaring et al., 2001; Kaufmann et al., 2021; Patterson et al., 1998). As previously shown, stress responses as well as a history of stressors such as previous injury were strongly associated with injury rates in dancers (van Winden et al., 2021) and other athletes (Andersen and Williams, 1988; Ivarsson et al., 2017). We, however, showed the reverse association and that the injury as such should be regarded as a stressor for the dancer since the aftermath of an injury results in heightened stress levels and thus can lead to less optimal or reduced ability to perform or, in a worst-case scenario, the need to quit training and performing until fully recovered.

Injuries come with emotional, physiological, cognitive, and social ramifications, which potentially increase stress reactions in the injured (Mainwaring and Krasnow, 1999; Brewer, 1994; Leddy et al., 1994; Mainwaring et al., 1993; Kellezi et al., 2017). Injuries leave a person with pain as well as a variety of interchanging emotions. These emotions range from shock to anxiety, confusion, worry, frustration up to optimism and are depending on the severity of the injury and external influences (Mainwaring, 1999) as well as prospect and success of rehabilitation (Mainwaring et al., 2001). Our results showed that our dancers can be compared to other athletes, because both show increases of stress during injury periods (Putukian, 2016).

However, differences between dancers and other athletes should be noted. Research in sports medicine has shown that first time injured athletes experienced the rehabilitation period as more stressful, they were less self-confident and exhibited a lower overall mood compared to subsequently injured athletes (Johnson, 1996). In contrast, subsequently injured athletes, however, coped better with second or third injury than with first time injuries. They were more accepting of subsequent injuries, were more socially secure and less anxious and thus likely less stressed (Johnson, 1996). This is not in agreement with our findings in dancers. We found that stress levels after subsequent injuries did not differ from stress levels after first injuries. Two aspects, namely the handling of injury in traditional dance settings as well as the resulting negative outcomes and thus negative experience of first injury might explain this difference and are discussed in the following.

Sports science has documented the so-called athletic identity which describes the tendency in athletes to continue training despite injury and pain, specifically in those athletes whose identification with their sport and dedicated discipline, i.e., athletic identity, was very high (Weinberg et al., 2013). In dance, the same tendency was found which can be detrimental to dancers’ health (Wainwright et al., 2005; Turner and Wainwright, 2003; Jacobs et al., 2017). In sports medicine (Mainwaring, 1999), injury is regarded as a threat to athletes’ and institutions’ existence. Dancers live in fear of injury and pain (Wainwright et al., 2005). Simultaneously, however, they also have to learn to accept injuries as a part of their dance careers and work through them to avoid sanctions or missing out on opportunities (Wainwright et al., 2005; Karreman et al., 2019; Kaufmann et al., 2021). While many dancers perceive the confusion resulting from this counter-intuitive approach (Wainwright et al., 2005), they try to accept it as part of their identity and attempt to regard injury as a positive sign of vocation, dedication and discipline in order to cope (Wainwright et al., 2005; Kelman, 2000).

However, there is a fine line between working through and accepting injury (Harrison and Ruddock-Hudson, 2017; Putukian, 2016) versus failing to show self-responsible health seeking behavior which includes seeking assistance, getting diagnosis and treatment and rehabilitating injury to avoid further consequences such as overuse or subsequent injury (Mackian et al., 2004). This ill-advised hardiness as coping strategy after an injury could contribute to the increased stress response our dancers also and especially perceived after subsequent injury.

Furthermore, and comparable to other sports professionals (Tesarz et al., 2012), dancers exhibited higher pain and pain tolerance thresholds (Tajetfoxell and Rose, 1995) than non-athletic controls. A variety of biological as well as psychological factors introduced through rigorous professional athletic training are considered to be responsible (Tesarz et al., 2012). However, in contrast to other athletes, dancers were also found to be more acutely sensitive to pain (Tajetfoxell and Rose, 1995). Although this appears to be a paradox on first thought, the study tried to explain it through dancers’ high experience of pain and injury as well as their fear of it (Tajetfoxell and Rose, 1995). Those results and our results underscore the importance of adequate handling of the first injury, since inadequate handling could result in problematic stress responses as well as negative outcomes of injury which both can negatively affect healing and prolong recovery (Noh et al., 2005; Mainwaring et al., 1993; Kellezi et al., 2017; Noh et al., 2009; American College of Sports, M, et al., 2006). As such, dancers’ specifically high stress levels after subsequent injury might result from such acute sensitivity as a consequence of their fear of negative experiences after their first injury.

### 4.1 Practical implementations and future research

Our results showed that dancers perceive elevated stress levels during first and subsequent injury periods. Above all, implementing primary injury prevention measures in order to spare dancers such stress experiences cannot be overstated. Furthermore, adequate handling of pain and injury in dance settings have been emphasized in literature before (Jenkins et al., 2013; Harrison and Ruddock-Hudson, 2017; Tajetfoxell and Rose, 1995). Our results show that it is essential to help dancers to cope with their stress during periods of injury; however, they also emphasize the importance to actively support stress management in dancers in the first place. Various reasons might contribute to the elevated stress response levels which need to be investigated in order to be able to assist dancers in finding ways of reducing and handling those stress responses. Among them are adequate coping skills of the dancer (van Winden et al., 2020a), but also active compliance to primary injury prevention as well as health seeking behavior in case of injury. The latter includes reporting injuries (Karreman et al., 2019) which needs to be fostered through empowering motivational climates (Kaufmann et al., 2021), emotional and social support (Patterson et al., 1998) as well as the importance of and compliance to holistic injury rehabilitation taking not only the injury but also psychosocial aspects into account (Mainwaring, 1999).

Diagnosis, treatment and rehabilitation of injuries implies coming to terms with the injury and its sequelae, including the application of coping skills and thus building resilience. While moving through multidimensional phases of injury appraisal through targeted rehabilitation (Mainwaring, 1999), emotions in athletes have shown to be changing from predominantly negative at first to becoming more positive, particularly after diagnosis (Clement et al., 2015). Reasons are that the knowledge somebody gains through diagnosis will decrease injury-related stress levels and thus coping behavior (Mainwaring and Krasnow, 1999). Going through rehabilitation allows one to reflect on the lessons learned through injury occurrence (Clement et al., 2015). As such, they increase resilience and are essential for future coping behavior but also secondary injury prevention. If dancers, for whatever reason, deny and continue training at the pre-injury level they might remain engulfed in negative emotions by missing out on those beneficial effects of diagnosis and rehabilitation. Further research is needed to investigate whether this might increase stress levels and make them prone to a subsequent injury.

### 4.2 Strengths, limitations and recommendations

To our knowledge this is the first study to investigate injury as a stressor in dancers. A major strength of this study is the longitudinal prospective design. Nonetheless, some limitations have to be addressed. The self-reported injuries and stress scores led to limited diagnostic details available for analysis. Future research could combine objective with subjective documentation of injuries and stress perception, including medical diagnosis of injuries as well as stress measurement based on physical parameters such as amylase and cortisol sampling (Berndt et al., 2012; Takai et al., 2004). Furthermore, we assessed stress on a monthly basis, and therefore, short periods of stress peaks (van der Does et al., 2017) as well as likely daily fluctuations (von Rosen et al., 2017) might be overlooked. Moreover, we cannot say whether stress levels and injury occurrence were an exact timely match, since we did not record stress on a day-to-day basis. Also, part of the effect we found could be due to seasonal fluctuations of stress levels, for instance due to exams. Using higher sampling frequencies, for instance weekly, will provide more accurate data, although compliance might be lower (Richardson et al., 2017). Future studies should prospectively assess injury as a stressor in various dance populations as well as in bigger sample sizes, since we ended up with small sample sizes due to stratification, in order to be able to conclude for different age groups, dance styles, dance levels and years of dance experience.

## 5 Conclusion

Our results suggest that injury in itself can be regarded as a stressor in dancers. As such, our study emphasizes the importance to invest in education for dancers which would enhance their coping and stress management skills. Such skills are important to prevent injuries resulting from higher stress levels, but also to facilitate the handling of stress following an injury occurrence. Moreover, injury rehabilitation programs should include the teaching of specific coping skills as well as knowledge on injury prevention for dancers themselves. Both are needed to come to terms with an injury that has already occurred, but also to be able to proactively prevent subsequent injuries after rehabilitation. Finally, primary injury prevention programs for dancers as well as their compliance are needed to protect dancers from pain, injury and related high stress levels in the first place.

## Data Availability

The raw data supporting the conclusions of this article will be made available by the authors, without undue reservation.
